# Total Saponins from Rhizoma Panacis Majoris Promote Wound Healing in Diabetic Rats by Regulating Inflammatory Dysregulation

**DOI:** 10.3390/ijms27020955

**Published:** 2026-01-18

**Authors:** Xiang Xu, Mei-Xia Wang, Ya-Ning Zhu, Xiang-Duo Zuo, Di Hu, Jing-Ping Li

**Affiliations:** 1 Faculty of Chinese Medicine, Yunnan University of Chinese Medicine, Kunming 650000, China; xxrrqqcn123@163.com (X.X.);; 2 Faculty of Pharmacy, Kunming Medical University, Kunming 650000, China

**Keywords:** diabetic wounds, total saponins from Rhizoma Panacis majoris, inflammatory response, neutrophil extracellular traps, macrophage polarisation, Wnt/β-catenin

## Abstract

In individuals with diabetes, dysregulation of inflammatory processes hinders the progression of wounds into the proliferative phase, resulting in chronic, non-healing wounds. Total saponins from Rhizoma Panacis majoris (*SRPM*), bioactive compounds naturally extracted from the rhizome of *Panax japonicus C.A.Mey. var. major (Burk.) C.Y.Wu and K.M.Feng*, have demonstrated extensive anti-inflammatory and immunomodulatory properties. This study aims to elucidate the molecular mechanisms underlying the facilitative effects of *SRPM* on diabetic wound healing, with particular emphasis on its anti-inflammatory actions. A high-fat diet combined with streptozotocin (STZ) administration was used to induce type 2 diabetes in rats. After two weeks of oral treatment with *SRPM* suspension, a wound model was established. Subsequently, a two-week course of combined local and systemic therapy was administered using both *SRPM* suspension and *SRPM* gel. *SRPM* markedly reduces the levels of pro-inflammatory mediators, including IL-1α, IL-1β, IL-6, MIP-1α, TNF-α, and MCP-1, in both rat tissues and serum. Concurrently, it increases the expression of anti-inflammatory cytokines such as IL-10, TGF-β1, and PDGF-BB, while also enhancing the expression of the tissue remodelling marker bFGF. Additionally, *SRPM* significantly decreases the accumulation of apoptotic cells within tissues by downregulating the pro-apoptotic gene *Caspase-3*, upregulating the anti-apoptotic gene *Bcl-2*, and increasing the expression of the apoptotic cell clearance receptor MerTK. Moreover, *SRPM* inhibits neutrophil infiltration and the release of neutrophil extracellular traps (NETs) in tissues, promotes macrophage polarisation towards the M2 phenotype, and activates the Wnt/β-catenin signalling pathway at the molecular level. *SRPM* promotes the healing of wounds in diabetic rats potentially due to its anti-inflammatory properties.

## 1. Introduction

Cutaneous manifestations associated with diabetes mellitus (DM), whether arising spontaneously or due to external factors, represent some of the most common and complex clinical complications. Notable examples include diabetic ulcers, peripheral neuropathy, and blister formation [1]. According to the 11th edition of the International Diabetes Federation (IDF) Global Diabetes Map, the point prevalence of diabetic foot ulcers among adults with diabetes is reported to be 6.3%, corresponding to an estimated 40 million individuals worldwide. Moreover, the cumulative lifetime risk of developing a foot ulcer in this population reaches up to 25%. These conditions impose considerable economic strain on affected patients and constitute a significant social and healthcare challenge [2].

The proper initiation, progression, and resolution of inflammation during wound healing are critical determinants for the successful transition of tissue into the proliferative and remodelling phases [3]. In contrast, diabetic patients exhibit impaired neutrophil function, resulting in excessive and sustained production of neutrophil extracellular traps (NETs) [4]. This phenomenon not only causes direct tissue damage but also activates immune cells to secrete elevated levels of pro-inflammatory mediators. Additionally, NETs hinder macrophage-mediated phagocytosis of apoptotic cells, thereby maintaining macrophages in the pro-inflammatory M1 phenotype and obstructing their transition to the anti-inflammatory M2 phenotype [5]. The consequent accumulation of apoptotic cells leads to the release of damage-associated molecular patterns (DAMPs), which further amplify NET production, establishing a self-perpetuating cycle of chronic inflammation [6,7]. This mechanism is a principal contributor to the impaired wound healing observed in diabetic individuals [8].

The Wnt/β-catenin signalling pathway is a crucial regulatory mechanism in skin injury repair, significantly influencing both inflammatory and immune cell responses [9]. Evidence indicates that reduced Wnt/β-catenin activity impairs the regenerative capacity of keratinocytes and stem cells. Furthermore, diminished activity within this pathway negatively impacts the function of neutrophils and macrophages, as well as the efficiency of apoptotic cell clearance, thereby exacerbating inflammatory reactions [10]. Consequently, reactivating the Wnt/β-catenin pathway presents a promising therapeutic strategy for treating diabetic foot ulcers.

*Panax japonicus C.A.Mey. var. major (Burk.) C.Y.Wu and K.M.Feng* is a species within the genus Panax, belonging to the family Araliaceae. Total saponins from Rhizoma Panacis majoris (*SRPM*) comprise a group of saponin compounds derived from this plant, which constitute the principal pharmacological agents responsible for its therapeutic efficacy [11]. Previous studies have demonstrated that *SRPM* exhibits immunomodulatory, anti-inflammatory, and haematopoietic activities [12]. In the present study, type 2 diabetes was induced in rats through a combination of a high-fat diet and streptozotocin (STZ) administration, followed by the establishment of a wound model. The animals were subsequently treated with oral administration of *SRPM*, alongside topical application of *SRPM*G. Utilising UHPLC-Q Exactive technology, network pharmacology approaches, and molecular biological assays, we explored the modulatory effects of *SRPM* on inflammatory responses and immune dysregulation associated with diabetic wound healing.

This study aims to elucidate the molecular mechanisms underlying *SRPM* in the treatment of refractory wounds associated with diabetes mellitus, thereby providing a foundational basis for future research and development.

## 2. Results

### 2.1. Chemical Composition of SRPM

Mass spectrometric data for *SRPM* were acquired using UHPLC-Q Exactive instrumentation operated in both positive and negative ionisation modes (Supplementary Materials, Figure S2). The analysis indicated that the saponin constituents of *SRPM* exhibited an enhanced signal response in the negative ion mode. Data processing was performed using Progenesis QI version 3.0 software, with spectral matching conducted against the MJBIOTCM database. By integrating information from the TCMSP and PubChem databases, as well as relevant literature sources, a total of 38 saponins and sapogenins were identified (Table 1).

### 2.2. Target Molecules for SRPM Treatment of Diabetic Wounds

**Figure 1:**
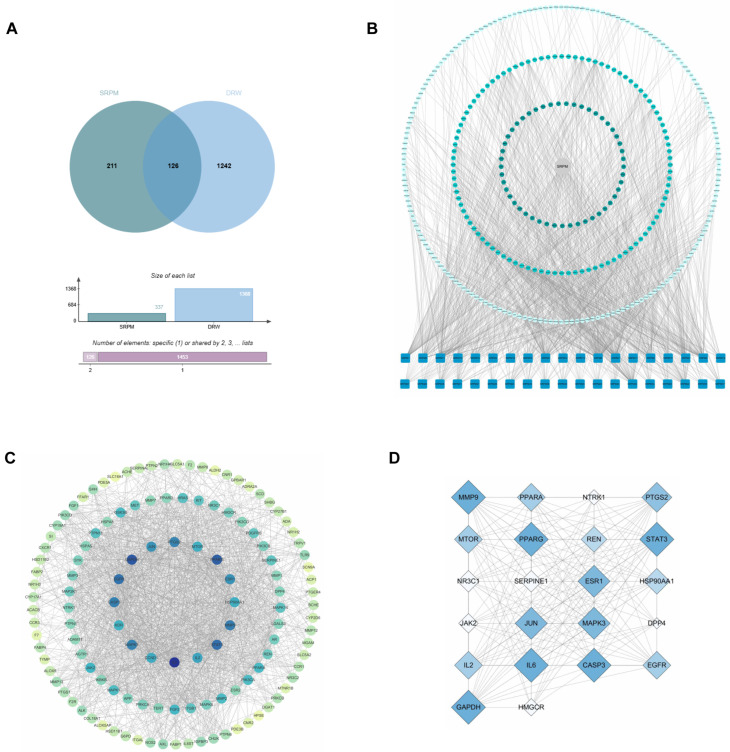
Prediction of *SRPM* target molecules. (**A**) Intersection of constituents and disease targets. (**B**) ‘Drug-Constituent-Target-Disease’ network relationships. (**C**) Protein–protein interaction (PPI) network. (**D**) Core targets screened from the PPI network (Betweenness > 110.9683, Closeness > 0.0043, Degree > 26.6191).

The SwissTargetPrediction database identified 337 potential target molecules associated with *SRPM*. From the GeneCards database, 5495 disease-related targets were retrieved, of which 1186 were selected based on a threshold score of ≥10. Additionally, the OMIM database contributed 241 disease-related targets. After integrating these datasets and removing duplicate entries, a total of 1368 candidate targets were obtained. The intersection of the component-related and disease-related target sets revealed 126 potential targets (Figure 1A). These targets were subsequently used to construct the ‘*SRPM*-component-target-disease’ network (Figure 1B). The 126 potential targets were further analysed using the STRING database to develop a protein–protein interaction (PPI) network, comprising 126 nodes and 1677 edges (Figure 1C). Network visualisation and analysis performed with Cytoscape 3.10.0 and the CentiScape 2.2 plugin identified 22 core targets within the network (Figure 1D).

### 2.3. GO and KEGG Enrichment Analysis

**Figure 2:**
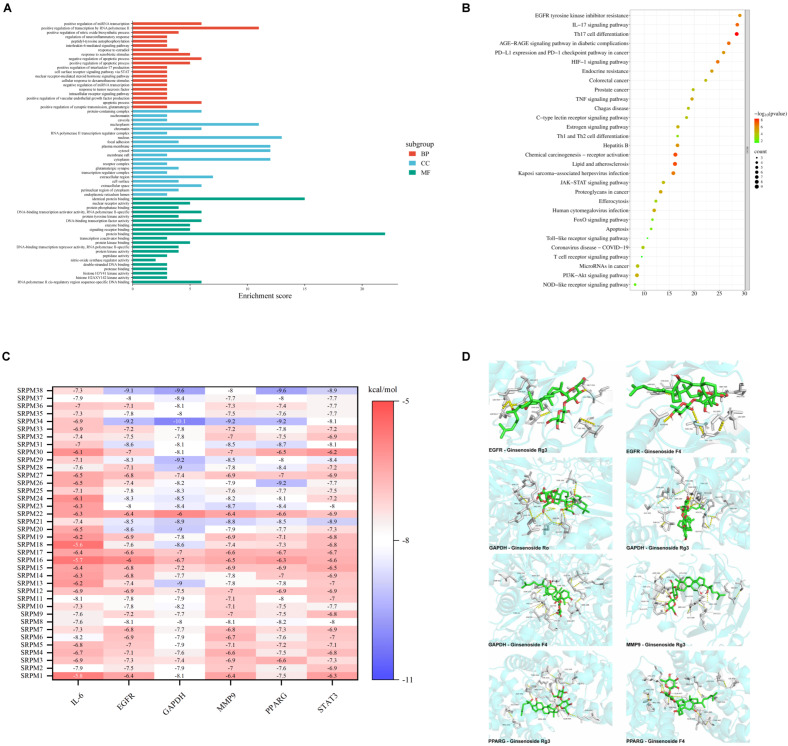
GO, KEGG enrichment analysis, and molecular docking. (**A**) GO enrichment analysis, top 20 entries. (**B**) KEGG pathway enrichment analysis, top 30 entries. (**C**) Molecule-target binding energies, all below −5 kcal/mol. (**D**) 3D visualisation of 8 optimal ‘molecule-target conformation’ pairs.

The 22 core targets implicated in the treatment of diabetic wounds by *SRPM* were subjected to Gene Ontology (GO) functional annotation and Kyoto Encyclopaedia of Genes and Genomes (KEGG) pathway analysis using the DAVID database. The GO enrichment analysis (Figure 2A) revealed that the biological processes (BPs) predominantly associated with *SRPM* intervention include the positive regulation of interleukin-17 production, the interleukin-6 signalling pathway, responses to tumour necrosis factor, and apoptosis. The key cellular components (CCs) identified encompass organelles, membrane structures, the cytoskeleton and cellular junctions, extracellular domains, and protein complexes. Regarding molecular functions (MFs), the principal activities involve protein binding, protein phosphatase activity, transcription co-activator functions, and signal receptor binding. KEGG pathway enrichment analysis (Figure 2B) demonstrated that the principal signalling pathways engaged by *SRPM* in the context of diabetic wound treatment comprise IL-17, TNF, JAK-STAT, AGE-RAGE, PI3K-Akt, NOD-like receptor, FoxO, and T cell receptor pathways. These pathways are fundamentally linked to processes of inflammation, immune regulation, as well as cellular proliferation and differentiation. Of particular interest, the enrichment of the efferocytosis pathway suggests that *SRPM* also modulates the phagocytic clearance of apoptotic cells, thereby contributing to immune homeostasis and tissue repair mechanisms.

### 2.4. Molecular Docking

The six highest-ranking targets, determined by their degree values, were identified from the core targets and designated as key targets. These key targets underwent molecular docking analysis using AutoDock 1.5.7 Vina with 38 chemical constituents of *SRPM* to calculate their binding energies (Figure 2C). All ligand–target interactions exhibited binding energies lower than −5 kcal/mol, indicating a favourable binding affinity [13]. Furthermore, the eight most optimal docking conformations were selected for three-dimensional visualisation (Figure 2D).

### 2.5. SRPM Promotes Wound Healing in Diabetic Rats

**Figure 3:**
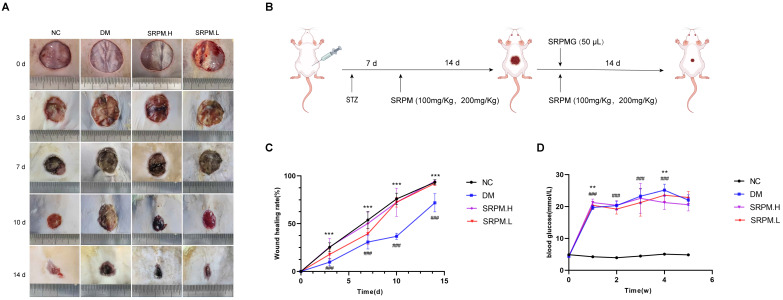
Effects of *SRPM* on wound healing rates and blood glucose levels in DM Rats. (**A**) Wound healing images at postoperative days 0, 3, 7, 10, and 14. (**B**) STZ-induced diabetic rat model pretreated with *SRPM* for 14 days; wound model established followed by consecutive 14-day intervention with oral *SRPM* (100, 200 mg/kg) + topical *SRPM*G (50 μL). (**C**) Wound healing rates at postoperative days 0, 3, 7, 10, and 14. Wound healing rate = (Original wound area − Unhealed wound area)/Original wound area × 100%. (**D**) Blood glucose changes in DM rats. *SRPM*.H: ‘200 mg/kg *SRPM*’ + ‘50 μL/rat *SRPM*G’; *SRPM*.L: ‘100 mg/kg *SRPM*’ + ‘50 μL/animal *SRPM*G’. Bar and line graphs represent mean ± standard deviation, *n* = 8 per group. DM vs. NC, ^###^ *p* < 0.001. *SRPM* vs. DM, ** *p* < 0.01; *** *p* < 0.001.

**Figure 4:**
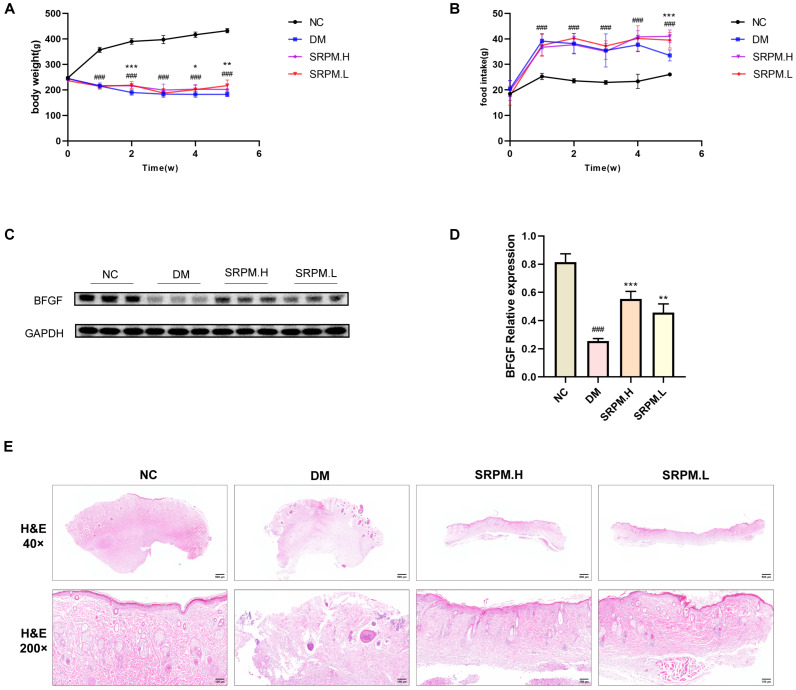
Effects of *SRPM* on body weight, food intake, and histopathology in DM Rats. (**A**,**B**) Changes in body weight and food intake in DM rats. (**C**) Representative Western blot bands for BFGF. (**D**) Expression levels of BFGF protein. (**E**) H&E staining: 40× scale bar = 500 μm; 200× scale bar = 100 μm. *SRPM*.H: ‘200 mg/kg *SRPM*’ + ‘50 μL/rat *SRPM*G’; *SRPM*.L: ‘100 mg/kg *SRPM*’ + ‘50 μL/animal *SRPM*G’. Bar and line graphs represent mean ± standard deviation, *n* = 8 per group. DM vs. NC, ^###^ *p* < 0.001. *SRPM* vs. DM, * *p* < 0.05; ** *p* < 0.01; *** *p* < 0.001.

Following establishment of the DM rat model, we administered *SRPM* via oral gavage for two weeks prior to surgical wound creation. Following surgery, the rats underwent a combined treatment regimen consisting of oral *SRPM* and topical application of *SRPM*G from day 0 to day 14 (Figure 3B). Compared to the DM control group, *SRPM* treatment significantly accelerated the rate of skin wound healing on days 3, 7, 10, and 14 (Figure 3A,C). Furthermore, rats receiving *SRPM* showed a significant reduction in blood glucose levels one week post-surgery (Figure 3D), as well as a notable increase in body weight both one week before surgery and during the first two weeks following surgery (Figure 4A).

Previous studies have suggested that basic fibroblast growth factor (bFGF) may serve as a biomarker for tissue remodelling [14]. In the present study, a significant downregulation of bFGF expression was observed in rats within the diabetic mellitus (DM) group. Conversely, bFGF expression was markedly upregulated in the *SRPM*-treated group compared to the DM group (Figure 4C,D).

Furthermore, histopathological changes in rat tissues were observed (Figure 4E). The DM group exhibited pronounced damage to the epidermal cell layer, accompanied by extensive infiltration of inflammatory cells in the adjacent regions. The deeper tissue layers showed widespread necrosis with an abundance of pus cells. In contrast, the normal control (NC) group displayed well-organised and tightly arranged epidermal layers, alongside prominent granulation tissue, collagen fibre deposition, and capillary proliferation within the dermis. In the high-dose *SRPM* group (*SRPM*.H), notable epidermal thickening was evident surrounding necrotic zones, with an orderly arrangement of collagen fibres, granulation tissue development, and enhanced vascularisation. The low-dose *SRPM* group (*SRPM*.L) demonstrated largely preserved epidermal integrity, while the dermis contained granulation tissue and neovascularisation characterised by compact tissue architecture.

### 2.6. SRPM Modulates Cytokine Levels to Alleviate Wound Inflammation

**Figure 5:**
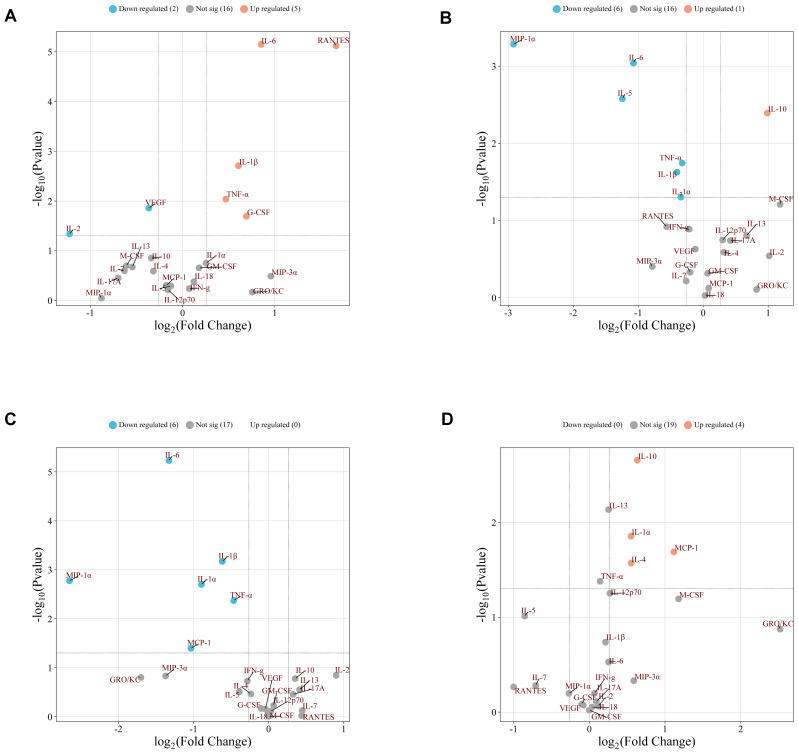
Intergroup differential expression analysis of cytokines within the tissue. (**A**) DM vs. NC yielded 7 differentially expressed proteins: G-CSF, IL-1β, IL-6, RANTES, TNF-α, IL-2, and VEGF. (**B**) *SRPM*.H vs. DM yielded 7 differentially expressed proteins: IL-10, IL-1α, IL-1β, IL-5, IL-6, MIP-1α, and TNF-α. (**C**) *SRPM*.L vs. DM yielded 6 differentially expressed proteins: IL-1α, IL-1β, IL-6, MCP-1, MIP-1α, and TNF-α. (**D**) *SRPM*.H vs. *SRPM*.L yielded 4 differentially expressed proteins: IL-10, IL-1α, IL-4, and MCP-1. Volcano plots display log_2_FC and −log_10_*p* on the x- and y-axes, respectively (log_2_FC ≥ 0 indicates upregulation, log_2_FC < 0 indicates downregulation).

Compared with the normal control (NC) group, the diabetic model (DM) group exhibited differential expression of seven proteins, characterised by upregulation of the pro-inflammatory cytokines G-CSF, IL-1β, IL-6, RANTES, and TNF-α, alongside downregulation of the anti-inflammatory factors IL-2 and VEGF (Figure 5A). Relative to the DM group, the *SRPM* treatment group showed seven differentially expressed proteins, with pro-inflammatory cytokines IL-1α, IL-1β, IL-6, MIP-1α, TNF-α, and MCP-1 downregulated, while the anti-inflammatory cytokine IL-10 was upregulated (Figure 5B,C). Furthermore, comparing the *SRPM* low-dose (*SRPM*.L) group with the high-dose (*SRPM*.H) group revealed four differentially expressed proteins, with the anti-inflammatory factors IL-10, IL-1α, IL-4, and MCP-1 all exhibiting increased expression in the *SRPM*.H group (Figure 5D).

**Figure 6:**
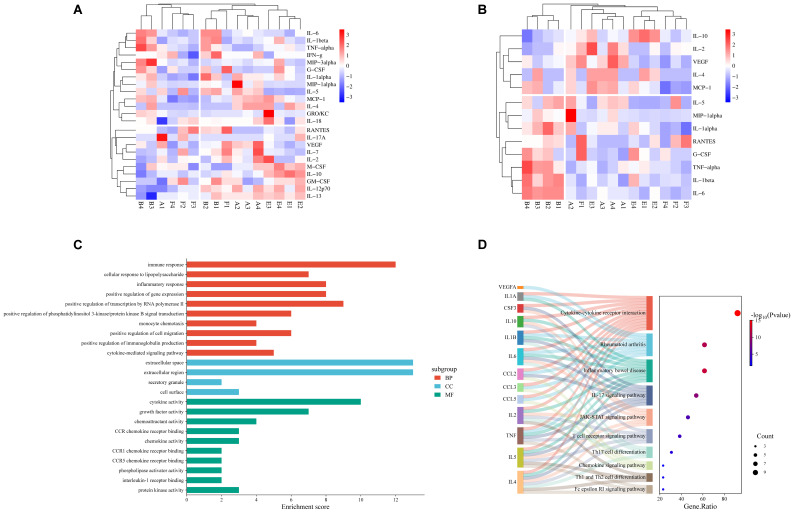
Bioinformatics analysis of differentially expressed cytokines in tissues. (**A**) Expression profiles of 23 cytokines across DM rat groups (*n* = 4 per group; A denotes NC group, B denotes DM group, E denotes *SRPM*.H group, and F denotes *SRPM*.L group). (**B**) Expression profiles of 13 intergroup differentially expressed proteins in DM rats across groups (FC > 1.2, *p* < 0.05). (**C**) GO enrichment analysis: BP and MF represent the top 10 entries, with CC having 4 entries. (**D**) KEGG pathway enrichment: top 10 entries.

We conducted a targeted analysis of the expression levels of 23 cytokines in rat skin tissue (Figure 6A). This screening identified 13 proteins with differential expression between groups: G-CSF, IL-1β, IL-6, RANTES, TNF-α, IL-2, VEGF, IL-10, IL-1α, IL-5, MIP-1α, MCP-1, and IL-4 (Figure 6B). Furthermore, Gene Ontology (GO) functional annotation and Kyoto Encyclopaedia of Genes and Genomes (KEGG) pathway analyses were performed on the 13 differentially expressed proteins identified in the tissue, using the DAVID database. The GO enrichment analysis (Figure 6C) revealed that the key biological processes (BPs) involved in *SRPM*-mediated regulation of cytokines include immune response, cellular response to lipopolysaccharide, inflammatory response, and cytokine-mediated signalling pathways. The predominant cellular components (CCs) associated with these proteins comprise the extracellular space and compartments, secretory granules (such as those containing hormones, enzymes, and neurotransmitters), and cell surface structures. Regarding molecular functions (MFs), the principal activities involve cytokine activity, growth factor activity, chemokine activity, phospholipase activation, and protein kinase activity. KEGG pathway enrichment analysis (Figure 6D) identified that the principal signalling pathways engaged in *SRPM*’s regulation of cytokines include Fc epsilon RI signalling, differentiation of Th1 and Th2 cells, chemokine signalling, Th17 cell differentiation, T cell receptor signalling, JAK-STAT signalling, and IL-17 signalling pathways.

**Figure 7:**
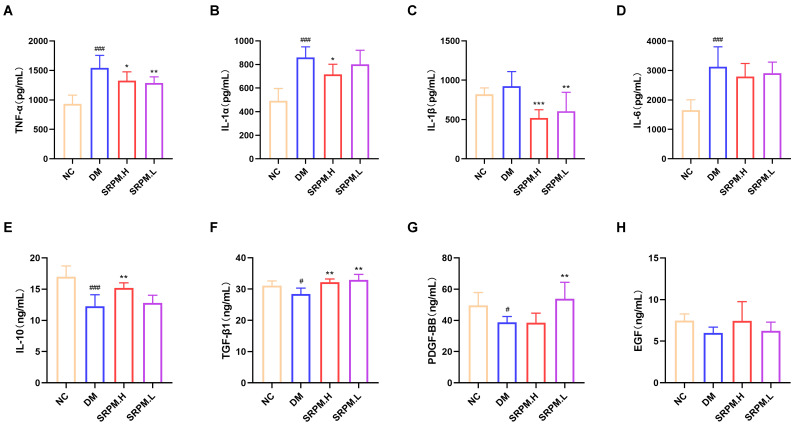
ELISA detection of cytokine levels in rat serum. (**A**–**D**) Expression levels of pro-inflammatory factors TNF-α, IL-1α, IL-1β, and IL-6. (**E**–**H**) Expression levels of anti-inflammatory factors IL-10, TGF-β1, PDGF-BB, and EGF. *SRPM*.H: ‘200 mg/kg *SRPM*’ + ‘50 μL/animal *SRPM*G’; *SRPM*.L: ‘100 mg/kg *SRPM*’ + ‘50 μL/animal *SRPM*G’. Bar charts represent mean ± standard deviation, *n* = 6 per group. DM vs. NC, ^#^ *p* < 0.05; ^###^ *p* < 0.001. *SRPM* vs. DM, * *p* < 0.05; ** *p* < 0.01; *** *p* < 0.001.

Finally, cytokine concentrations were measured in the serum of rats. The findings indicated that, compared with the diabetic model (DM) group, treatment with *SRPM* markedly reduced the serum levels of the pro-inflammatory cytokines TNF-α, IL-1β, and IL-1α in diabetic rats. Simultaneously, *SRPM* administration led to a significant increase in the concentrations of the anti-inflammatory cytokines IL-10, TGF-β1, and PDGF-BB (Figure 7A–H).

### 2.7. SRPM Effectively Reduces Abnormal Accumulation of Apoptotic Cells

**Figure 8:**
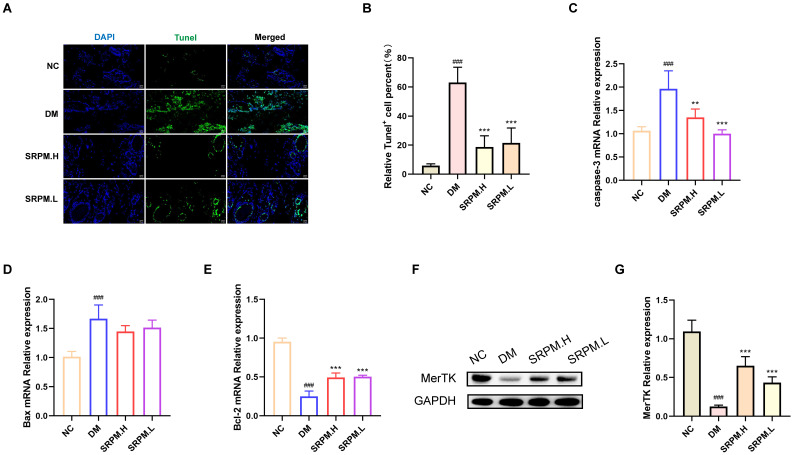
Detection of apoptotic cell numbers and related molecular levels in DM rat tissues. (**A**) TUNEL staining (green fluorescence) for apoptotic cells. (**B**) Statistical analysis of apoptotic cell proportion in tissues. (**C**–**E**) RT-qPCR detection of mRNA expression levels for apoptotic genes *Caspase-3* and *Bax*, and anti-apoptotic gene *Bcl2* in tissues. (**F**) Representative Western blot bands for MerTK. (**G**) MerTK protein expression levels. *SRPM*.H: ‘200 mg/kg *SRPM*’ + ‘50 μL/animal *SRPM*G’; *SRPM*.L: ‘100 mg/kg *SRPM*’ + ‘50 μL/animal *SRPM*G’. Bar charts represent mean ± standard deviation, *n* = 4 per group. Fluorescence scale bar: 20 μm. DM vs. NC: ^###^ *p* < 0.001. *SRPM* vs. DM: ** *p* < 0.01; *** *p* < 0.001.

A marked increase in apoptotic cell accumulation was observed in the tissues of diabetic mellitus (DM) rats. Notably, treatment with *SRPM* resulted in a significant reduction in the number of apoptotic cells in the tissues of DM rats compared with the untreated DM group (Figure 8A,B). Concurrently, *SRPM* administration led to a significant upregulation of *Bcl2* mRNA expression and a downregulation of *Caspase-3* mRNA expression in the tissues of DM rats (Figure 8C–E). Furthermore, *SRPM* treatment was found to significantly enhance MerTK expression levels in the tissues of DM rats (Figure 8F,G).

### 2.8. SRPM Inhibits Neutrophil Recruitment and Excessive NET Release

**Figure 9:**
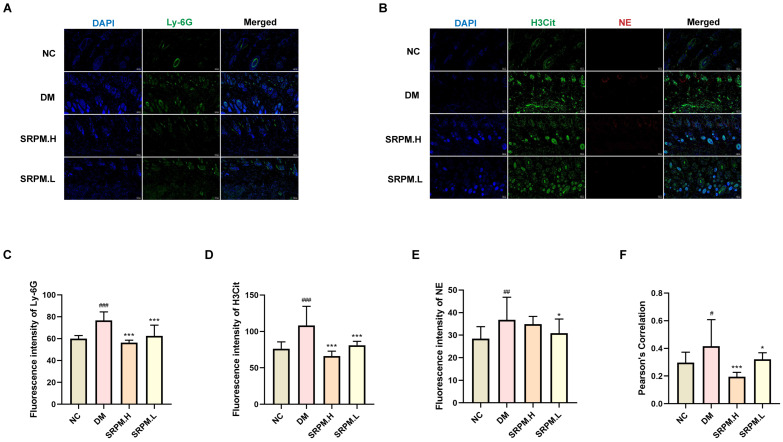
Immunofluorescence detection of neutrophil and NET quantity and distribution in rat skin tissue. (**A**) Ly-6G (green fluorescence) specifically labels neutrophils. (**B**) H3Cit (green fluorescence) and NE (red fluorescence) jointly label NETs. (**C**–**E**) Quantitative statistics of Ly-6G, H3Cit, and NE expression levels. (**F**) Pearson colocalisation coefficient statistics for H3Cit and NE (approaching 1 indicates higher spatial overlap of fluorescence intensities; approaching −1 indicates lower overlap). *SRPM*.H: ‘200 mg/kg *SRPM*’ + ‘50 μL/animal *SRPM*G’; *SRPM*.L: ‘100 mg/kg *SRPM*’ + ‘50 μL/animal *SRPM*G’. Bar charts represent mean ± standard deviation, *n* = 4 per group. Fluorescence scale bar: 100 μm. DM vs. NC, ^#^ *p* < 0.05; ^##^ *p* < 0.01; ^###^ *p* < 0.001. *SRPM* vs. DM, * *p* < 0.05; *** *p* < 0.001.

The results of the study demonstrated a significant increase in Ly-6G fluorescence expression within the skin tissue of rats in the diabetic mellitus (DM) group. Conversely, treatment with *SRPM* in DM rats resulted in a marked reduction in Ly-6G fluorescence expression in the skin tissue (Figure 9A,C). Additionally, *SRPM* administration led to a significant decrease in the fluorescence expression of H3Cit and NE in the skin tissue of these rats (Figure 9B,D,E). Notably, the Pearson correlation coefficient between H3Cit and NE fluorescence signals approached −1, indicating minimal spatial colocalisation between the two markers (Figure 9F).

### 2.9. SRPM Promotes the Conversion of Macrophages from M1 to M2 Type

**Figure 10:**
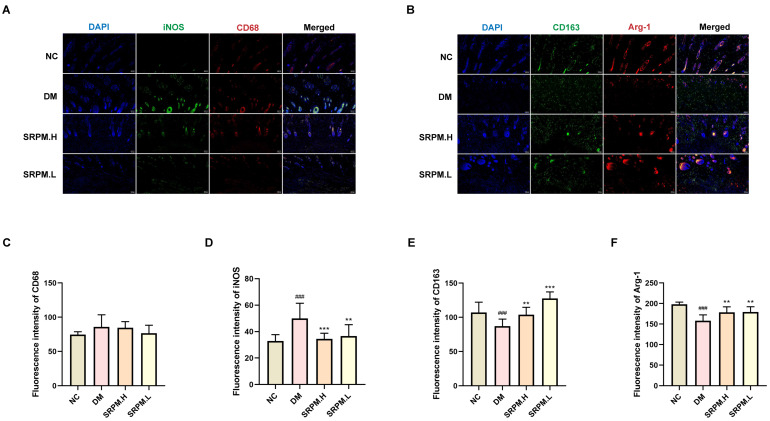
Immunofluorescence detection of M1 and M2 macrophage numbers and distribution in rat skin tissue. (**A**) M1 macrophages co-labelled with iNOS (green fluorescence) and CD68 (red fluorescence). (**B**) CD163 (green fluorescence) and Arg-1 (red fluorescence) jointly mark M2 macrophages. (**C**–**F**) Quantitative analysis of CD68, iNOS, CD163, and Arg-1 expression levels. *SRPM*.H: ‘200 mg/kg *SRPM*’ + ‘50 μL/animal *SRPM*G’; *SRPM*.L: ‘100 mg/kg *SRPM*’ + ‘50 μL/animal *SRPM*G’. Bar charts represent mean ± standard deviation, *n* = 4 per group. Fluorescence scale bar: 100 μm. DM vs. NC, ^###^ *p* < 0.001. *SRPM* vs. DM, ** *p* < 0.01; *** *p* < 0.001.

The results of the study demonstrated a marked increase in the fluorescent expression of inducible nitric oxide synthase (iNOS) within the tissues of rats in the diabetic mellitus (DM) group. Conversely, rats treated with *SRPM* showed a significant reduction in iNOS fluorescence expression, while the fluorescence expression of CD68 remained unchanged (Figure 10A,C,D). Additionally, the DM group exhibited a significant decrease in the fluorescent expression of CD163 and arginase-1 (Arg-1). Treatment with *SRPM* resulted in a significant increase in the fluorescence expression levels of both CD163 and Arg-1 (Figure 10B,E,F).

### 2.10. SRPM Activates the Wnt/β-Catenin Pathway to Modulate Inflammation and Immune Dysregulation

**Figure 11:**
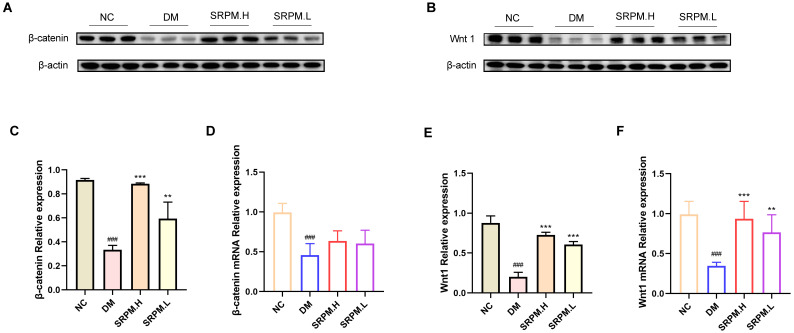
Expression of molecules related to the Wnt/β-catenin pathway and analysis of inflammation in rat skin tissue. (**A**) Representative Western blot band for β-catenin. (**B**) Representative Western blot band for Wnt1. (**C**) β-catenin protein expression levels. (**D**) *β-catenin* mRNA expression levels. (**E**) Wnt1 protein expression levels. (**F**) Expression levels of *Wnt1* mRNA. *SRPM*.H: ‘200 mg/kg *SRPM*’ + ‘50 μL/animal *SRPM*G’; *SRPM*.L: ‘100 mg/kg *SRPM*’ + ‘50 μL/animal *SRPM*G’. Bar charts represent mean ± standard deviation, *n* = 4 per group. DM vs. NC, ^###^ *p* < 0.001. *SRPM* vs. DM, ** *p* < 0.01; *** *p* < 0.001.

A significant downregulation of Wnt1 and β-catenin protein expression was observed in the tissues of rats in the diabetic mellitus (DM) group. Conversely, rats treated with *SRPM* demonstrated a marked upregulation of Wnt1 and β-catenin protein levels compared to the DM group (Figure 11A–C,E). Additionally, analysis revealed that *Wnt1* mRNA expression was significantly elevated in the *SRPM*-treated group, whereas *β-catenin* mRNA levels did not show a statistically significant change (Figure 11D,F).

**Figure 12:**
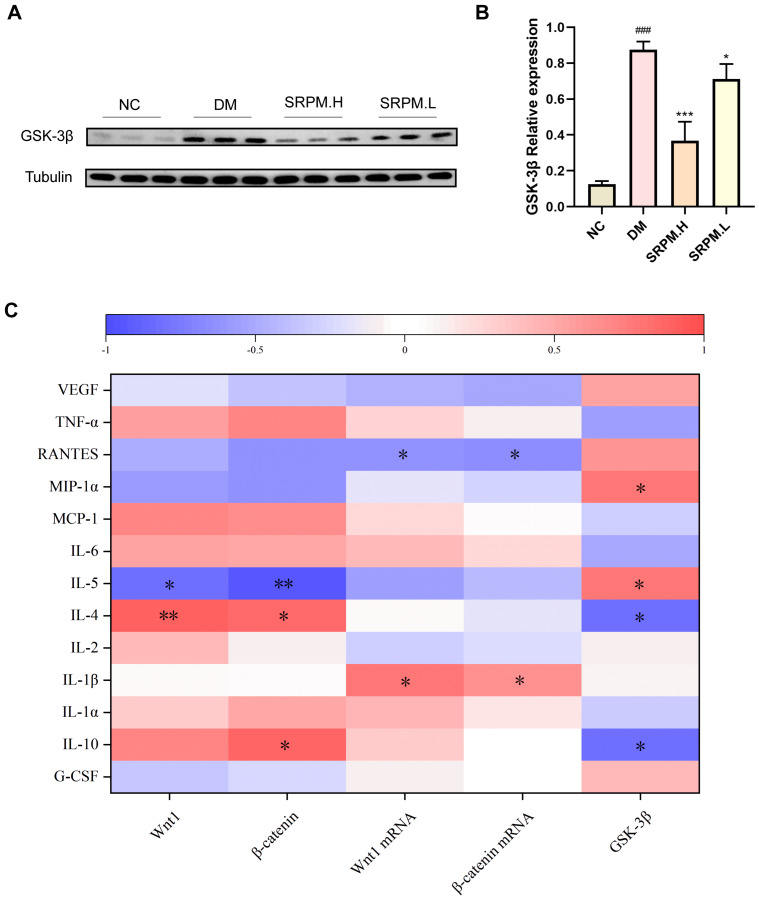
Expression levels of GSK-3β in rat tissues and Pearson correlation analysis. (**A**) Representative Western blot bands for GSK-3β. (**B**) Protein expression levels of GSK-3β. (**C**) Pearson correlation coefficient analysis between Wnt/β-catenin pathway expression and cytokine levels (0–1 indicates positive correlation, −1–0 indicates negative correlation; ** *p* < 0.01, * *p* < 0.05). *SRPM*.H: ‘200 mg/kg *SRPM*’ + ‘50 μL/animal *SRPM*G’; *SRPM*.L: ‘100 mg/kg *SRPM*’ + ‘50 μL/animal *SRPM*G’. Bar charts represent mean ± standard deviation, *n* = 4 per group. DM vs. NC, ^###^ *p* < 0.001. *SRPM* vs. DM, * *p* < 0.05; *** *p* < 0.001.

Furthermore, our analysis revealed a significant upregulation of GSK-3β protein expression in the diabetic mellitus (DM) group. Conversely, GSK-3β protein levels were markedly downregulated in tissue samples obtained from DM rats treated with *SRPM* (Figure 12A,B). Finally, the results indicated significant correlations between the expression levels of components within the Wnt/β-catenin signalling pathway and various cytokines, including IL-10, IL-4, IL-5, IL-1β, MIP-1α, and RANTES (Figure 12C).

## 3. Discussion

The pathogenesis of diabetic wounds involves an intertwined dual axis of systemic and local factors. Such conditions prove difficult to manage effectively through topical dressing changes alone or hypoglycaemic agents alone; simultaneous intervention targeting both systemic metabolism and the local wound site is essential. Our preliminary pre-experiments revealed that (Supplementary Materials, Table S1; Figure S3) topical application of *SRPM* alone significantly accelerated wound healing. Conversely, while oral administration of *SRPM* markedly improved glycaemic control, wound healing rates showed no statistically significant difference compared to the model group. Consequently, we fixed the topical dosage and employed a gastric administration with a dose gradient to verify whether the marked improvement in blood glucose levels could synergise with topical *SRPM* application, yielding dose-dependent additional healing benefits. Indeed, *SRPM*.H demonstrated a further enhancement in wound healing rates compared to *SRPM*.L. However, upon examining the underlying mechanisms, this dose–response relationship proved difficult to reconcile. Specifically, the active components of *SRPM* may undergo metabolism within the body before exerting their effects. The concentration and distribution of these metabolites do not correlate simply with the original drug dosage. Furthermore, systemic therapeutic effects and local therapeutic effects overlap in terms of timing, targets, and pathways. Consequently, the dosage levels of systemic administration struggle to establish a stepwise dose–response relationship at the molecular level.

In diabetic wounds, immune dysregulation and excessive inflammation together create a vicious cycle, ultimately resulting in delayed wound healing and the development of chronic wounds under diabetic conditions [15]. Currently, FDA-approved bioactive drugs for diabetic wounds have limitations, including a limited range, inconsistent efficacy, significant side effects, and poor tolerability. Research has shown that *SRPM* possesses anti-inflammatory, analgesic, and immunomodulatory properties [16], which align closely with the core pathogenesis of hard-to-heal diabetic wounds.

The UHPLC-Q Exactive system utilises electrostatic field Orbitrap technology, delivering a resolution of up to 140,000 FWHM. This allows for the effective separation of compounds within complex samples, facilitating the rapid, simultaneous identification and characterisation of multiple constituents, even in the absence of reference standards [17]. Using UHPLC-Q Exactive liquid chromatography–mass spectrometry analysis, we predicted and identified 38 saponins and sapogenins from *SRPM* in both positive and negative ion modes. Concurrently, our network pharmacology predictions suggest that *SRPM* may exert its therapeutic effects on diabetic wounds by modulating inflammatory responses and cellular immune functions. Finally, we selected the top six receptor proteins with the highest degree values from the core target set as key targets. Molecular docking with all 38 chemical constituents of *SRPM* demonstrated favourable binding affinities.

Within a hyperglycaemic environment, immune cell dysfunction occurs, leading to excessive production of pro-inflammatory cytokines and insufficient secretion of anti-inflammatory cytokines [18]. This cytokine imbalance further exacerbates the inflammatory response, hindering the transition of the wound into the proliferative phase and consequently resulting in chronic, non-healing wounds [19]. This study investigated the combined treatment of diabetic mellitus (DM) rat wounds using oral *SRPM* and topical *SRPM*G application. The results demonstrated that *SRPM* upregulates basic fibroblast growth factor (bFGF) at the molecular level, promoting cell proliferation and tissue repair, thereby significantly improving the histopathological morphology of DM rats. We further employed Luminex liquid-phase suspension chip technology to target and screen seven cytokines from the Bio-Plex Pro Rat 23-Cytokine Panel associated with *SRPM*’s regulation of inflammation in diabetic wounds. The results showed that *SRPM* downregulated pro-inflammatory cytokines, including IL-1α, IL-1β, IL-6, MIP-1α, TNF-α, and MCP-1 in rat skin tissue, while upregulating the IL-10 expression, thereby partially correcting the cytokine imbalance. Concurrently, ELISA results demonstrated that *SRPM* also downregulated pro-inflammatory cytokines TNF-α, IL-1β, and IL-1α in rat serum, while upregulating anti-inflammatory cytokines IL-10, TGF-β1, and PDGF-BB, thus ameliorating the persistent inflammatory state. Ren et al. demonstrated in a rheumatoid arthritis model that RPMTG (i.e., *SRPM*) restores synovial pathology by suppressing IL-1β/IL-6 and activating the p38 MAPK and PI3K/Akt/mTOR pathways [20]. This corroborates the ‘anti-inflammatory-promoting repair’ effect observed in diabetic wounds in the present study, collectively revealing *SRPM*’s core mechanism in regulating inflammation and tissue repair across diseases.

In diabetic wounds, there is a significant positive correlation between the accumulation of apoptotic cells and excessive inflammation [21], which together create a chronic inflammatory microenvironment [22,23]. MerTK (Mer tyrosine kinase), a member of the TAM family (TYRO3, AXL, MERTK), is one of the most critical receptors involved in efferocytosis. It primarily mediates phagocytosis by indirectly recognising phosphatidylserine (PS) on the surface of apoptotic cells [24]. Through TUNEL staining, we observed that *SRPM* significantly reduced apoptotic cell accumulation in diabetic rat wounds. Potential mechanisms may include upregulating the anti-apoptotic gene *Bcl-2*, downregulating the pro-apoptotic gene *Caspase-3*, and enhancing the expression of the apoptotic cell clearance receptor MerTK. This synergistically inhibits apoptosis while accelerating the clearance of apoptotic cells. Pang et al. reported in rheumatoid arthritis (RA) synovium that RPMTG (*SRPM*) reduces HMGB1, enhances Beclin-1–*Bcl-2* binding, and induces apoptosis in RA-FLS [25]. Thus, *SRPM* is not a simple ‘pro-apoptotic’ or ‘anti-apoptotic’ agent, but rather ‘resets’ the apoptosis–autophagy balance according to the pathological microenvironment. Research indicates that persistent hyperglycaemia promotes nuclear-to-cytoplasmic translocation of HMGB1, which acts as a signalling molecule to activate the ERK/Ets-1 pathway, indirectly elevating the *Bcl2*/*Bcl-2* ratio [26]. Inhibiting HMGB1 via shRNA blocks this cascade, significantly reducing hyperglycaemia-induced cardiomyocyte apoptosis. This suggests that *SRPM* may suppress HMGB1 levels in the hyperglycaemic microenvironment, thereby reversing pro-apoptotic effects and offering a direction for future investigations.

Furthermore, in diabetic patients, impaired glucose metabolism leads to excessive activation and recruitment of neutrophils following skin injury, resulting in an overproduction of neutrophil extracellular traps (NETs) [27]. This process impedes macrophage phagocytosis of apoptotic cells, causing macrophages to persist in the pro-inflammatory M1 phenotype and hindering their polarisation towards the anti-inflammatory M2 phenotype [28]. Consequently, this drives and sustains excessive inflammatory responses at the wound site [29]. The present experimental findings demonstrate that *SRPM* significantly reduces the expression of Ly-6G, neutrophil elastase (NE), and citrullinated histone H3 (H3Cit) in the wound tissues of diabetic rats. This confirms *SRPM*’s capacity to inhibit excessive neutrophil migration and recruitment to sites of skin injury, thereby diminishing NET formation. We further observed a marked decrease in the expression of the M1-specific marker inducible nitric oxide synthase (iNOS) and a significant increase in the expression of the M2-specific markers arginase-1 (Arg-1) and CD163 within the wound tissues of diabetic rats. Relevant studies have confirmed that TSPJ (*SRPM*) downregulates mRNA and protein expression of Ly6G, Mac-2, and cyclooxygenase-2 (COX-2) in liver tissue, thereby inhibiting neutrophil infiltration and macrophage recruitment [30]. The present wound model experiment extends these findings by demonstrating suppression of upstream events governing NET formation and neutrophil recruitment. This further corroborates *SRPM*’s anti-inflammatory properties, wherein its cellular targets adapt to tissue and pathological environments. In summary, *SRPM* ameliorates inflammatory responses by reducing apoptotic cell accumulation, inhibiting neutrophil NET release, and synergistically promoting macrophage polarisation towards the M2 phenotype.

The Wnt/β-catenin signalling pathway induces macrophage polarisation towards the M2 anti-inflammatory phenotype, upregulating anti-inflammatory factors such as IL-10 and TGF-β1, while inhibiting persistent neutrophil infiltration and NET release [31]. In diabetic wounds, reduced Wnt activity traps macrophages in the M1 state, impeding the clearance of apoptotic cells and leading to excessive inflammatory responses [32]. Existing research indicates that β-catenin signalling directly drives M2 macrophage polarisation: nuclear β-catenin stabilisation inhibits the NF-κB–IL-12 axis while simultaneously upregulating M2-characteristic factors such as IL-10, TGF-β, and Arg-1 [33]; blocking β-catenin/TCF transcription (using ICG001) markedly attenuates IL-4-induced M2 polarisation [34]. Conversely, this pathway suppresses excessive NET formation, thereby reducing the clearance burden at its source. Activation of Wnt/β-catenin reduces neutrophil PAD4 and p38 MAPK activity, decreasing high-glucose-induced NET production by 30–50% and restoring endothelial β-catenin/TCF4 signalling suppressed by NETs. This reactivates DNase1L3 transcription, enhancing local DNA degradation capacity [35,36]. Typically, within the Wnt/β-catenin signalling pathway, GSK-3β maintains low levels of free intracellular β-catenin by phosphorylating it, thereby promoting its degradation via the ubiquitin-proteasome system [37]. Consequently, GSK-3β serves as a pivotal regulatory brake within the Wnt/β-catenin pathway, with its activity directly determining β-catenin stability and pathway activation. The present experimental findings demonstrate that *SRPM* significantly upregulates Wnt1 and β-catenin expression in rat skin tissue, while concurrently downregulating levels of GSK-3β, a molecule promoting β-catenin phosphorylation. Therefore, the potential mechanism by which *SRPM* modulates inflammation and immune dysregulation in diabetic wounds may be associated with the activation of the Wnt/β-catenin signalling pathway.

## 4. Materials and Methods

### 4.1. Reagents

Carbomer 940, glycerol, and triethanolamine were purchased from Yien Chemical Technology Co., Ltd. (Shanghai, China); ethyl paraben was obtained from Yuanye Biotechnology Co., Ltd. (Shanghai, China). Chromatography-grade methanol, acetonitrile, formic acid, and acetic acid were acquired from Fisher Chemical (Waltham, MA, USA); mass spectrometry grade water, 2-Propanol, and 2-Chloro-L-Phenylalanine were obtained from Merck (Rahway, NJ, USA). Streptomycin (STZ) was purchased from Sigma (St. Louis, MO, USA); USP-grade sodium carboxymethylcellulose was obtained from Aladdin Biochemical Technology Co., Ltd. (Shanghai, China).

### 4.2. Preparation of SRPM and SRPMG

Dried rhizomes of Panacis majoris were sourced from Lijiang, China (Batch No.: 20241005) and identified by the Department of Chinese Materia Medica Identification at Yunnan University of Chinese Medicine as the dried rhizomes of Panacis majoris, belonging to the Araliaceae family and the genus Panax. Precisely weigh 1 kg, grind, then add to 10 L of 70% ethanol solution. Soak for 2 h, reflux extract three times, combine extracts, and reduce under vacuum until no ethanol odour remains. Subsequently, add threefold volume of water-saturated n-butanol for extraction, 1 h per cycle, repeated four times; combine extracts. Evaporate to dryness under reduced pressure again, dissolve in 1 L methanol, and filter. Add 4–5 volumes acetone to the filtrate, shake thoroughly, and stand for 4 h. Filter under suction, rinse the residue with appropriate acetone 2–3 times, and dry the residue in an 80 °C oven for 2 h. This yielded 82 g of white powdered *SRPM*, with a calculated yield of 8.2% (Supplementary Materials Figure S1). Following methods referenced in the 2020 edition of the Chinese Pharmacopoeia and the relevant literature [38,39], the mass fraction of *SRPM* was determined to be 78% by UV2700 ultraviolet-visible spectrophotometer (Shimadzu, Kyoto City, Japan).

Dissolve 2 g of *SRPM* in 20 mL of 0.5% sodium carboxymethylcellulose buffer solution to form Phase A. Take 2.5 g of Carbomer 940 and dissolve in 50 mL of deionised water, allowing it to swell overnight. Take 16.1 g of this solution to form Phase B. Combine Phase A with Phase B and stir using a magnetic stirrer. Sequentially add 2.6 g glycerol, 0.4 g triethanolamine, and 0.015 mL 5% hydroxybenzoate ethanol. Stir thoroughly for 15 min to obtain a pale yellow *SRPM* gel (*SRPM*G) (Supplementary Materials Figure S1). Calculations indicate that each 1 µL of *SRPM* gel contains 0.05 mg of *SRPM*. Separately, prepare 20 mL of 0.5% sodium carboxymethyl cellulose buffer solution as Phase A. Follow the remaining steps as above to obtain the blank gel (CBCM).

### 4.3. SRPM Component Characterisation

Accurately weigh 1 mg of *SRPM* powder, dissolve in 1 mL of methanol solution, and sonicate at 35 °C for 10 min (40 kHz, 300 W). Filter through a 0.22 μm membrane filter to obtain the test solution.

Using a UHPLC-Q Exactive system (Thermo Scientific, Waltham, MA, USA) coupled with ultra-high-performance liquid chromatography and tandem Fourier transform ion-pair mass spectrometry, the chromatographic column employed was an ACQUITY UPLC BEH C18 (100 mm × 2.1 mm i.d., 1.7 µm; Waters, Milford, CT, USA); mobile phase A: 2% acetonitrile in water (containing 0.1% formic acid); mobile phase B: acetonitrile (containing 0.1% formic acid). Injection volume: 3 μL. Column temperature: 40 °C. Elution programme: 0–2 min, 5–20% B; 2–5 min, 20–35% B; 5–10 min, 35–65% B; 10–15 min, 65–95% B; 15–17 min, 95–5% B.

The sample underwent electrospray ionisation, with mass spectrometry signals acquired in both positive and negative ion scanning modes. Scan range (*m*/*z*): 70–1050; sheath gas flow rate: 50 arb; auxiliary gas flow rate: 13 arb; heating temperature: 450 °C; capillary temperature: 320 °C; positive mode ionisation voltage: 3500 V; negative mode ionisation voltage: −3000 V; S-Lens voltage: 40 V; collision energy (%): 20, 40, 60; MS full-scan resolution: 70,000; MS^2^ scan resolution: 17,500.

Baseline filtering, peak identification, integration, retention time correction, and peak alignment were performed using ProgenesisQI v3.0 software (Waters Corporation, Milford, CT, USA). Subsequently, characteristic peaks were searched against a database for identification. MS and MS/MS mass spectrometry data were matched against the Metabolite Database for Traditional Chinese Medicine (MJBIOTCM), with the MS mass error set to less than 10 ppm. Compounds were preliminarily identified based on secondary mass spectrometry matching scores. Chemical constituents related to Rhizoma Panacis majoris and the Panax genus were identified by searching the TCMSP database (https://tcmsp-e.com/tcmspsearch.php, accessed on 15 June 2025), PubChem database (https://pubchem.ncbi.nlm.nih.gov, accessed on 20 June 2025), and the published literature. Compounds were further confirmed by integrating actual ion fragmentation patterns, cleavage behaviour, and chromatographic retention characteristics [40].

### 4.4. Network Pharmacology Analysis

#### 4.4.1. SRPM Target Prediction

Potential action targets for chemical constituents in *SRPM* were collected using the PubChem and SwissADME (http://www.swissadme.ch/) retrieval platforms, alongside the SwissTargetPrediction (swisstargetprediction.ch) database. Using ‘Diabetic wound’ as the keyword, disease targets associated with diabetic wounds were collected from the GeneCards (https://www.genecards.org/) and OMIM (https://www.omim.org/) databases, then merged, with duplicates removed. The Venny 2.1.0 online platform was employed to determine the intersection between *SRPM* component actions and potential disease targets. The resulting intersecting genes were then imported into Cytosccape 3.10.0 software to visually represent the complex relationships among *SRPM* components, targets, and diseases.

#### 4.4.2. Bioinformatics Analysis

The intersection genes between *SRPM* components and diseases were imported into the STRING database (https://cn.string-db.org/), with the species set to Homo sapiens (combined score > 0.4), to obtain the protein–protein interaction (PPI) network. The TSV format file was downloaded and imported into Cytoscape 3.10.0 software, where visualisation analysis was performed using the Centiscape 2.2 plugin. Based on the core targets identified through PPI visualisation, data were imported into the DAVID database (https://davidbioinformatics.nih.gov/), with the species set to Homo sapiens for Gene Ontology (GO) and KEGG pathway enrichment analyses, selecting entries with *p*-values < 0.05. GO encompasses analysis across three dimensions: biological process (BP), cellular component (CC), and molecular function (MF). KEGG primarily focuses on analysing signal transduction pathways involved in drug-mediated disease treatment processes [41]. Integrating these findings, we explored the potential mechanisms by which *SRPM* may exert therapeutic effects on diabetic wounds, providing clear direction for subsequent experimental validation.

#### 4.4.3. Molecular Docking Validation

The 2D structural diagram of the active ingredient *SRPM* was downloaded from the PubChem database. Using Chem3D 23.1.1 software, the structure underwent minimisation processing and was converted into a 3D model. Protein crystal structures of key targets were obtained from the UniPort and RCSB PDB databases, then imported into PyMOL 3.2 software for processing including dehydration and ligand removal. Molecular docking was performed using Autodock Vina between the small-molecule ligands and protein receptors to calculate binding energies. This identified potential key active components, which were then visualised using PyMOL.

### 4.5. Experimental Animals

Thirty-two healthy male SD rats, aged 4 weeks and weighing (160 ± 20) g, were procured from Beijing Huafukang Biotechnology Co., Ltd. (Beijing, China). Production Licence No.: SCXK (Jing) 2020-0004; Use Licence No.: SYXK (Yunnan) K2022-0004. Rats were housed in the SPF-grade animal facility at Yunnan University of Chinese Medicine, with 24 h access to food and water at room temperature under optimal humidity conditions. All animal experiments were conducted in accordance with the Guidelines for the Care and Use of Laboratory Animals. This study was approved by the Animal Experiment Ethics Review Committee of Yunnan University of Chinese Medicine, with ethics review number: R-062024G017.

### 4.6. Model Establishment and Treatment

Twenty-four rats were randomly selected and fed a high-fat diet for 4 weeks. Following 16 h of fasting without water restriction, they received intraperitoneal injections of STZ (prepared at 10 mg/mL in 0.1 mol/L sodium citrate buffer) at a dose of 30 mg/kg. Eight healthy control rats (NC) received an equal volume of sodium citrate buffer solution via intraperitoneal injection. After 72 h, rats exhibiting fasting blood glucose levels ≥ 11.0 mmol/L, alongside the ‘three excesses and one deficiency’ symptoms (excessive eating, excessive drinking, excessive urination, and weight loss), were confirmed as type 2 diabetic rat models.

DM rats were randomly assigned to the following groups: untreated DM group (DM), high-dose *SRPM* treatment group (*SRPM*.H), and low-dose *SRPM* treatment group (*SRPM*.L), with 8 rats per group. The *SRPM*.H and *SRPM*.L groups received oral administration of *SRPM* suspension at 200 mg/kg and 100 mg/kg, respectively, while the NC and DM groups received an equal volume of distilled water (3 mL). Following 2 weeks of oral treatment, anaesthesia was induced via intraperitoneal injection of sodium pentobarbital (40 mg/kg). Back hair was shaved, and a 2 cm diameter sterile circular plastic disc was used to mark and excise the full thickness of skin without damaging the subcutaneous fascia layer. The wound was then covered with surgical tape and secured with medical adhesive tape. Rats were housed individually with free access to food and water. One day preoperatively and two days postoperatively, penicillin (40,000 U/animal) was administered intramuscularly. Following the establishment of the DM wound model, in addition to continued gastric lavage treatment, the *SRPM* group received topical application of 50 μL/animal *SRPM*G; the NC and DM groups received topical application of 50 μL/animal CBCM. Gastric lavage and wound dressing changes were performed once daily for 14 consecutive days of intervention.

On day 14 post-surgery, rats were anaesthetised via intraperitoneal injection of sodium pentobarbital (40 mg/kg). Arterial blood samples were collected and stored at −80 °C. Skin tissue samples were obtained from a 2 mm perimeter around the wound site, divided into two portions, fixed in 4% paraformaldehyde solution, and stored at −80 °C.

### 4.7. Haematoxylin and Eosin Staining

Tissue samples from wound margins, fixed in 4% paraformaldehyde solution, were dehydrated, cleared, and embedded in paraffin to produce sections. Sections were dewaxed to water, followed by haematoxylin staining, acid-alcohol differentiation, eosin staining, clearing, and mounting. Under microscopic examination, re-epithelialisation, inflammatory cell infiltration, granulation tissue growth, and collagen fibre arrangement were assessed.

### 4.8. Luminex Liquid-Phase Suspension Chip

Precisely weigh the skin tissue and transfer it to a 2 mL centrifuge tube after washing. Add 100 µL RIPA (medium) lysis buffer and homogenise thoroughly using a tissue homogeniser at 60 Hz for 120 s. Process using an ultrasonic homogeniser for 1 min, cycling ultrasonication for 2 s followed by 5 s on ice, repeated for 1 min. Allow to stand on ice for 30 min post-ultrasonication. Centrifuge at 13,200 rpm at 4 °C for 15 min. Transfer the supernatant to a centrifuge tube. Quantify protein concentration using the BCA Protein Concentration Assay Kit (Proteintech, Rosemont, IL, USA) according to the manufacturer’s protocol.

Weigh the skin tissue, centrifuge at 10,000 rpm for 10 min using the sample tissue lysis buffer, collect the supernatant, and perform equal-mass detection at 45 µg. Dilute with Sample Diluent: RIPA (medium) at a 24:1 ratio, supplemented with 0.5% BSA, to a final volume of 50 µL. Employ the Bio-Plex Pro Rat 23-plex Cytokine Group I Panel antibody array (Bio-Rad, Hercules, CA, USA). Proceed sequentially according to the operating manual: standard dilution, sample incubation (1 h), antibody detection incubation (30 min), PE incubation (10 min), followed by colour development. Detection was performed using the Luminex 200 suspension bead chip platform (Luminex, Austin, TX, USA). Fluorescence signals were automatically calculated and optimised by software, generating output files in Excel format. Intergroup differences in cytokine levels across groups were analysed using IBM SPSS Statistics 27 software. Proteins exhibiting intergroup differences were selected based on criteria of (Fold Change, FC) > 1.2 and (*p*-value, *p*) < 0.05, followed by bioinformatics analysis.

### 4.9. Enzyme-Linked Immunosorbent Assay (ELISA)

ELISA kits for tumour necrosis factor-α (TNF-α), interleukin-1β (IL-1β), interleukin-1α (IL-1α), interleukin-6 (IL-6), interleukin-10 (IL-10), Transforming Growth Factor-β1 (TGF-β1), Epidermal Growth Factor (EGF), and Platelet-Derived Growth Factor-BB (PDGF-BB) were procured from Feiyue Biotechnology Co., Ltd. (Wuhan, China). Following the kit instructions, serum levels of pro-inflammatory factors TNF-α, IL-1β, IL-1α, and IL-6 and anti-inflammatory factors IL-10, TGF-β1, EGF, and PDGF-BB were sequentially measured in DM-induced skin lesion rats. Readings were obtained using a SpectraMax Plus microplate reader (Molecular Devices, San Jose, CA, USA), generating an output file in Excel format.

### 4.10. TUNEL Staining

Immerse the tissue sections twice in xylene, followed by two washes with absolute ethanol. Wash sequentially in 95%, 70%, and 50% ethanol for 3 min each. Wash samples twice with PBS, add 20 µg/mL proteinase K solution, and incubate for 15 min. Following kit instructions, add TUNEL staining reagent and incubate at 37 °C for 2 h under light-protected conditions. Counterstain with DAPI for 10 min, mount slides, and examine under a fluorescence microscope.

### 4.11. Immunofluorescence

Rat skin tissue was fixed in 4% paraformaldehyde for 24 h, dehydrated using a gradient ethanol series, and subsequently embedded in paraffin. Embedded tissue was sectioned into 3–5 μm thick slices and mounted onto microscope slides. The skin tissue sections were sequentially placed in eco-friendly dewaxing solution I, II, and III for 10 min each, followed by anhydrous ethanol I, II, and III for 5 min each, and finally rinsed with distilled water. After antigen retrieval, the sections were allowed to cool naturally. The slides were then placed in PBS (pH 7.4) and washed three times on a decolourisation shaker for 5 min each. After gently centrifuging the slides to remove excess solution, mark the tissue periphery with a histochemistry pen and add blocking solution (3% BSA) dropwise. Incubate for 30 min.

Neurophil Elastase Rabbit Polyclonal Antibody (NE) and Histone H3 Recombinant Rabbit Monoclonal Antibody (H3) were purchased from Hua’an Biotechnology Co., Ltd. (Hangzhou, China); Rabbit Anti-Arginase 1 Antibody, Rabbit Anti-CD163 Antibody, Rabbit Anti-CD68 Antibody, and Rabbit Anti-iNOS Antibody were purchased from Bio-Ocean Biotechnology Co., Ltd. (Beijing, China). Following the antibody incubation protocol (Table 2), add the primary antibody diluted 1:200. Place the slides flat in a humid chamber and incubate overnight at 4 °C. Transfer the slides to PBS (pH 7.4) and wash three times on a decolourisation shaker for 5 min each. Add the corresponding secondary antibody (diluted 1:150) and incubate at room temperature in the dark for 50 min. Place the slides in PBS (pH 7.4) and wash three times on a decolourisation shaker for 5 min each time. Add DAPI stain solution and incubate at room temperature in the dark for 10 min. Place the slides in PBS (pH 7.4) and wash three times, each for 5 min. Add autofluorescence quencher solution B, allow to act for 5 min, then rinse under running water for 10 min. Seal the slides with an antifluorescence-quenching sealing agent.

Fluorescence signals were observed and recorded using a Ts2R-FL inverted fluorescence microscope (Nikon Corporation, Tokyo, Japan). An average of four images per section were captured at 200× magnification, with Mean Fluorescence Intensity (MFI) calculated using Fiji-ImageJ 1.54 software. The Pearson colocalisation coefficient was calculated to analyse the co-localisation of H3 and NE within the tissue (a Pearson coefficient closer to 1 indicates higher spatial overlap of fluorescence intensities between the two markers, while a value closer to −1 indicates lower overlap).

### 4.12. Western Blot

Prepare separating and concentrating gels. Add loading buffer and boil for 5 min. After cooling, load 10 µL of sample. Employ a vertical electrophoresis apparatus (Beijing Liuyi Instrument Factory, Beijing, China) for 10% SDS-PAGE electrophoresis (80 V for 40 min, then 120 V for 80 min) until target bands are resolved. Transfer proteins to a PVDF membrane using a transfer apparatus (300 mA, 60 min). Block with 5% BSA at room temperature for 2 h. Incubate overnight at 4 °C with diluted primary antibodies: Anti-MerTK, Anti-Wnt1, Anti-β-catenin, Anti-GSK-3β, and Anti-bFGF (Affinity, San Francisco, CA, USA). Wash the membrane three times with 1× TBST (10 min/wash). Add Multi-rAb HRP-Goat Anti-Rabbit Recombinant secondary antibody (Proteintech, USA) and incubate at room temperature for 1 h. Wash the membrane. Detection was performed using the Ultra-Sensitive ECL Chemiluminescent Detection Kit (Proteintech, USA). ImageJ was employed to analyse grey values. Relative expression levels of MerTK, Wnt1, β-catenin, GSK-3β, and bFGF proteins were calculated using the ratio method after normalisation against the internal reference protein.

### 4.13. RT-qPCR

Following PBS washing of skin tissue, total RNA was extracted using the TriQuick Reagent Kit (Servicebio, Wuhan, China), with RNA concentration and purity assessed via SMA4000+V6.0.9. Programme settings: 25 °C for 5 min, 42 °C for 30 min, and 85 °C for 5 s. Reverse transcription to cDNA was performed using the Servicebio RT First Strand cDNA Synthesis Kit (Servicebio, Wuhan, China). Amplification programme settings: 95 °C for 30 s pre-denaturation (1×); 95 °C for 15 s denaturing; 60 °C for 30 s annealing/extending (40×). GAPDH served as the internal control. Results were analysed using the 2^−ΔΔCt^ method to calculate the relative expression levels of *Wnt1*, *β-catenin*, *Bax*, *Caspase-3*, and *Bcl2* mRNA. All primer sequences are detailed in Table 3.

### 4.14. Statistical Analysis

Statistical analysis was conducted using SPSS 27.0 software. Data are presented as mean ± standard deviation. Pairwise comparisons employed the two-sample LSD-t test, while multiple group comparisons utilised one-way analysis of variance (ANOVA). Where variances were unequal, pairwise *t*-tests were applied. Differences were considered statistically significant at *p* < 0.05.

## 5. Conclusions

In summary, as a natural saponin-derived active ingredient, *SRPM* precisely modulates the inflammatory immune network within diabetic wounds through a ‘multi-pathway, multi-target’ approach, thereby accelerating healing. Concurrently, this study employs an orthogonal model combining topical application with oral administration to delineate *SRPM*’s wound healing activity into distinct ‘local direct’ and ‘systemic indirect’ effects for the first time. This strategy establishes a universal evaluation paradigm for candidate drugs exhibiting both systemic and local activity. In the future, we will focus on analysing the spatiotemporal distribution of *SRPM*’s active ingredients and their metabolites, while advancing large animal validation and Phase I clinical dose exploration. This will establish the theoretical and data foundation for developing an integrated novel drug that simultaneously lowers blood glucose and promotes healing.

## 6. Patents

The work described herein has been submitted for patent application to the China National Intellectual Property Administration under the title: ‘Method for Preparing Total Saponins from Rhizoma Panacis majoris and Their Application in Preparing Medicinal Products for Difficult-to-Heal Diabetic Wounds’, Publication Number: CN120643607A).

## Figures and Tables

**Table 1:** *SRPM* chemical composition analysis.

NO.	Compound	Formula	Retention Time	Mode	Adducts	*m*/*z*	Error
*SRPM*1	Gentiopicrin	C_16_H_20_O_9_	3.63	pos	[M+H]	357.1151	−8.19
*SRPM*2	Soyasapogenol C	C_30_H_48_O_2_	5.44	pos	[M+H]	441.3721	−1.45
*SRPM*3	Ginsenoside F1	C_36_H_62_O_9_	5.87	pos	[M+H-2H_2_O]	603.4247	−1.26
*SRPM*4	Epibetulinic acid	C_30_H_48_O_3_	6.21	pos	[M+H]	457.3667	−2
*SRPM*5	2alpha,3beta,23-Trihydroxyolean-12-en-28-oic acid beta-D-glucopyranosyl ester	C_36_H_58_O_10_	6.65	pos	[M+H]	651.4152	7.63
*SRPM*6	Maslinic acid	C_30_H_48_O_4_	6.74	pos	[M+H-2H_2_O]	437.3407	−1.5
*SRPM*7	Corosolic acid	C_30_H_48_O_4_	7.13	pos	[M+H-2H_2_O]	437.3407	−1.48
*SRPM*8	Panaxatriol	C_30_H_52_O_4_	7.18	pos	[M+H-2H_2_O]	441.3725	−0.52
*SRPM*9	Hederagenin	C_30_H_48_O_4_	7.75	pos	[M+H-2H_2_O]	437.3408	−1.36
*SRPM*10	Pseudoginsenoside RT1	C_47_H_74_O_18_	9.84	pos	[M+NH_4_]	944.5197	−1.72
*SRPM*11	Oleanolic acid	C_30_H_48_O_3_	10.12	pos	[M+H-H_2_O]	439.3563	−1.68
*SRPM*12	Ginsenoside Rd	C_48_H_82_O_18_	10.83	pos	[M+Na]	969.5376	−1.83
*SRPM*13	Pseudoginsenoside Rc1	C_50_H_84_O_19_	11.28	pos	[M+Na]	1011.5484	−1.52
*SRPM*14	Ginsenoside CK	C_36_H_62_O_8_	12.2	pos	[M+H-2H_2_O]	587.4297	−1.53
*SRPM*15	Ginsenoside F2	C_42_H_72_O_13_	12.3	pos	[M+Na]	807.4849	−2.08
*SRPM*16	Zizybeoside I	C_19_H_28_O_11_	3.47	neg	[M-H]	431.1557	−0.4
*SRPM*17	Atractyloside G	C_21_H_36_O_8_	4.55	neg	[M+FA-H]	461.2395	0.61
*SRPM*18	Notoginsenoside R1	C_47_H_80_O_18_	5.25	neg	[M+FA-H]	977.5326	−0.12
*SRPM*19	Gypenoside XLVI	C_48_H_82_O_19_	5.45	neg	[M-H]	961.5374	−0.37
*SRPM*20	Ginsenoside Rg1	C_42_H_72_O_14_	5.53	neg	[M+FA-H]	845.4904	0.04
*SRPM*21	Majonoside R1	C_42_H_72_O_15_	5.75	neg	[M+FA-H]	861.4852	−0.17
*SRPM*22	Atractyloside A	C_21_H_36_O_10_	5.87	neg	[M-H]	447.2238	0.5
*SRPM*23	Majonoside R2	C_41_H_70_O_14_	6.23	neg	[M+FA-H]	831.475	0.31
*SRPM*24	Notoginsenoside R2	C_41_H_70_O_13_	7.88	neg	[M+FA-H]	815.4798	−0.08
*SRPM*25	Mogroside IIA	C_42_H_72_O_14_	8.02	neg	[M-H]	799.485	0.09
*SRPM*26	Ginsenoside Rf	C_42_H_72_O_14_	8.03	neg	[M+FA-H]	845.4907	0.37
*SRPM*27	Atractyloside D	C_27_H_46_O_12_	8.04	neg	[M+FA-H]	607.2976	0.8
*SRPM*28	Mogroside IIA1	C_42_H_72_O_14_	8.54	neg	[M-H]	799.4848	−0.12
*SRPM*29	Ginsenoside Ro	C_48_H_76_O_19_	9.17	neg	[M-H]	955.4905	−0.37
*SRPM*30	Ginsenoside Re	C_48_H_82_O_18_	9.83	neg	[M-H]	945.5415	−1.39
*SRPM*31	Chikusetsu saponin IVa	C_42_H_66_O_14_	9.94	neg	[M-H]	793.4376	−0.48
*SRPM*32	Ursolic acid	C_30_H_48_O_3_	10.57	neg	[M-H]	455.3531	0.02
*SRPM*33	Saikosaponin F	C_48_H_80_O_17_	10.83	neg	[M+FA-H]	987.5536	0.13
*SRPM*34	Ginsenoside Rg3	C_42_H_72_O_13_	12.22	neg	[M+FA-H]	829.4944	−1.38
*SRPM*35	Momordin IC	C_41_H_64_O_13_	12.57	neg	[M-H]	763.4275	0.08
*SRPM*36	Calenduloside E	C_36_H_56_O_9_	12.62	neg	[M-H]	631.3851	−0.05
*SRPM*37	Momordin Ib	C_36_H_56_O_9_	12.88	neg	[M-H]	631.3851	−0.14
*SRPM*38	Ginsenoside F4	C_42_H_70_O_12_	13.35	neg	[M+FA-H]	811.4847	−0.32

**Table 2:** Protocol for incubating antibodies in immunofluorescence.

Antibody	Target Cell/Protein	Sample
Rabbit Anti-Ly6g Antibody	Neutrophils	skin tissue
Neutrophil Elastase Rabbit pAb (NE)	Neu extracellular trap	skin tissue
Histone H3 Recombinant Rabbit Monoclonal Antibody (H3)		
Rabbit Anti-CD68 Antibody (CD68)	Total macrophages	skin tissue
Rabbit Anti-iNOS Antibody (iNOS)	M1-type macrophages (co-labelled with CD68)	skin tissue
Rabbit Anti-Arginase 1 Antibody (Arg-1)	M2-type macrophages	skin tissue
Rabbit Anti-CD163 Antibody (CD163)		

**Table 3:** Design of RT-qPCR primer sequences.

Gene	Primers	Sequence (5′ → 3′)	Length	Tm	GC%
*Wnt1*	Forward	TGGGGCATCGTGAACATAGC	20	60.46	55
	Reverse	GGTTCTGTCGGATCAGTCGT	20	59.47	55
*β-catenin*	Forward	GCTGAACCGTCACAGATGCT	20	56.35	55
	Reverse	GTCAGCTCAGGAATTGCACG	20	55.31	55
*Bax*	Forward	GAGACACCTGAGCTGACCTTG	21	59.5	57
	Reverse	GCTCCATGTTGTTGTCCAGTTC	22	57.7	50
*Caspase-3*	Forward	TTACCCTGAAATGGGCTTGTGT	22	60.16	45.45
	Reverse	TGAGGTTAGCTGCATCGACAT	21	59.52	47.62
*Bcl2*	Forward	AGAACTGCAGGTGCTGGATTTA	22	55.8	45
	Reverse	TAGATTTGTCTCCACAGCCACC	22	57.7	50
*GAPDH*	Forward	CTGGGCTACACTGAGCACC	19	55.46	63
	Reverse	AAGTGGTCGTTGAGGGCAATG	21	57.03	52

## Data Availability

The original contributions presented in this study are included in the article/Supplementary Materials. Further inquiries can be directed to the corresponding author.

## Supplementary Materials

The following supporting information can be downloaded at https://www.mdpi.com/article/10.3390/ijms27020955/s1.

## Abbreviations

The following abbreviations are used in this manuscript:

*SRPM*	Total Saponin from Rhizoma Panacis majoris
*SRPM*G	Gel preparation from Total Saponin extracted from Rhizoma Panacis majoris
DM	Diabetes Mellitus
GO	Gene Ontology
BP	Biological Process
CC	Cellular Component
MF	Molecular Function
KEGG	Kyoto Encyclopaedia of Genes and Genomes
PPI	Protein–Protein Interaction
