# Supplementary material for: Total Saponins from Rhizoma Panacis Majoris Promote Wound Healing in Diabetic Rats by Regulating Inflammatory Dysregulation
Source: Int J Mol Sci. 2026 Jan 18;27(2):955. doi: 10.3390/ijms27020955 (PMC12842158; doi:10.3390/ijms27020955)
Supplement: Supplementary file 1 [file ijms-27-00955-s001.zip › ijms-4089855-supplementary.pdf]

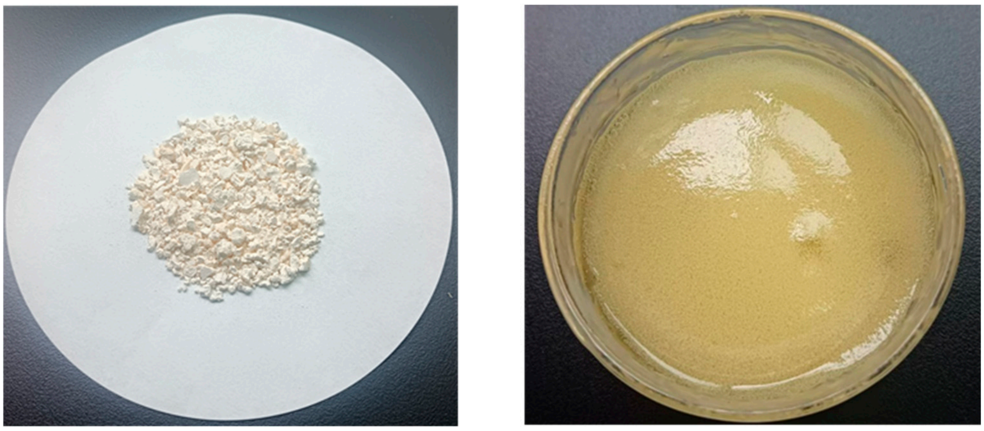

Figure S1. Image of Total Saponin from Rhizoma Panacis majoris and gel (SRPM & SRPMG) captured under a camera.

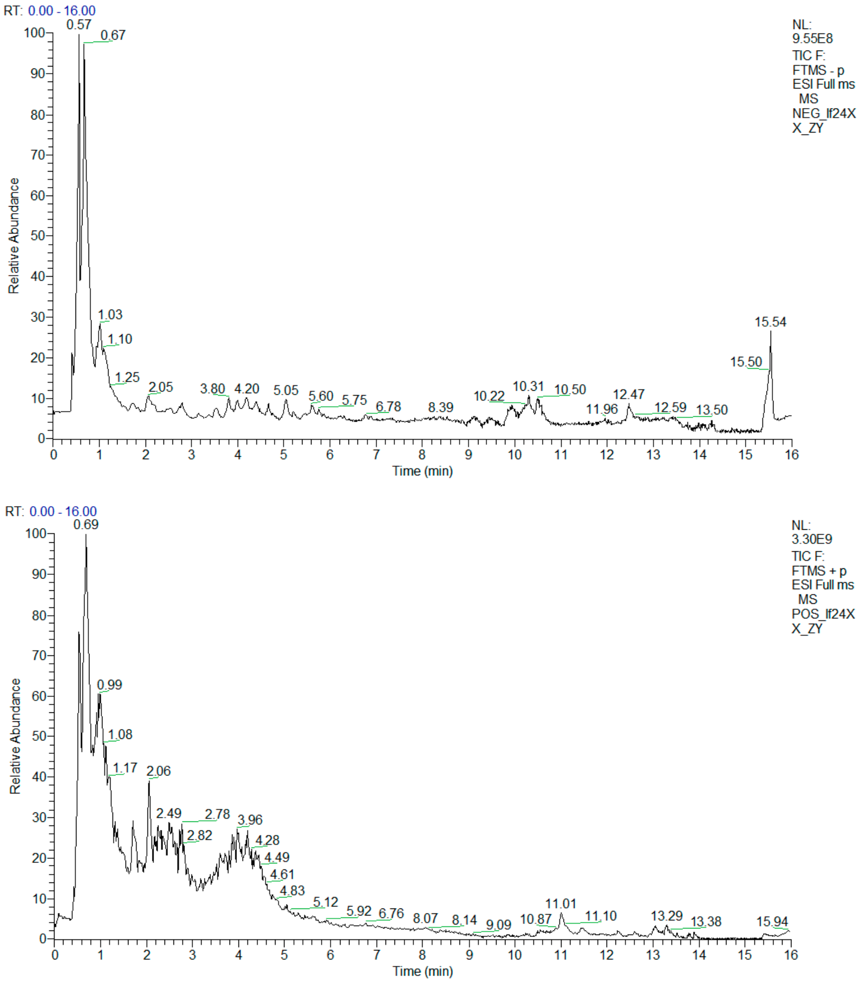

Figure S2. Total ion current chromatogram of SRPM in UHPLC-Q Exactive positive and negative ion modes.

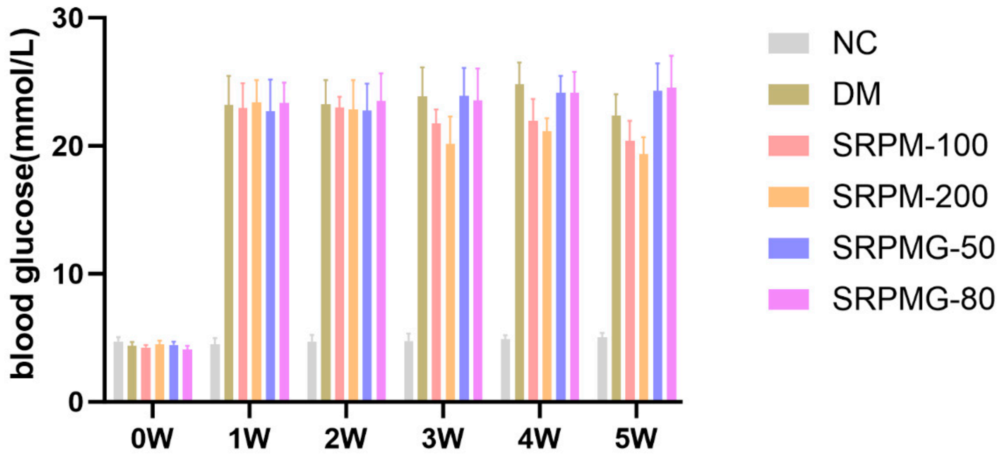

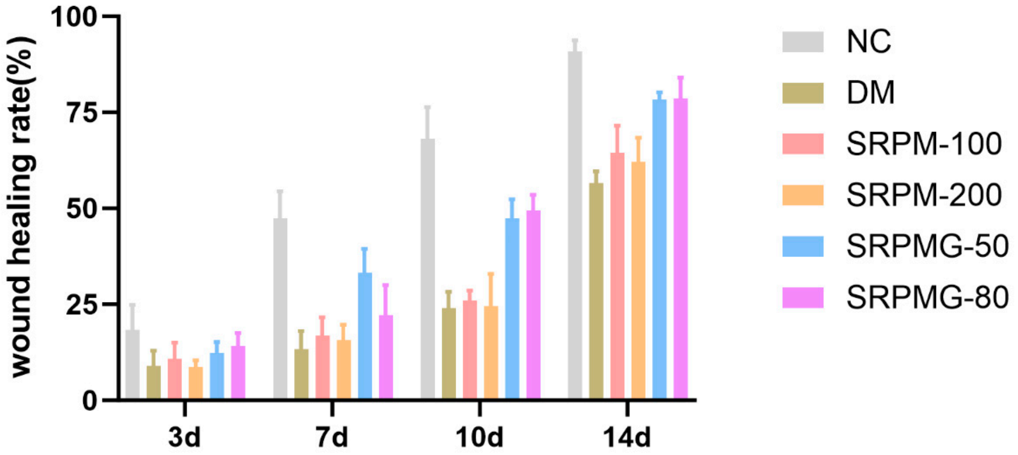

Figure S3. Effects of oral SRPM and topical SRPMG on blood glucose levels and wound healing rates in diabetic rats. SRPM-100: 100 mg/kg SRPM; SRPM-200: 200 mg/kg SRPM; SRPMG-50: 50 μL/rat SRPMG; SRPMG-80: 80 μL/rat SRPMG.

Table S1. Effects of SRPM Oral Administration and SRPMG Topical Application on Blood Glucose Levels and Wound Healing Rates in Diabetic Rats.

**indicator /Time**	**Group**					
	NC	DM	SRPM-100	SRPM-200	SRPMG-50	SRPMG-80
BG 0 W	4.7±0.34	4.38±0.28	4.25±0.17	4.48±0.28	4.47±0.24	4.08±0.28
BG 1 W	4.48±0.46	23.2±2.07	22.93±1.79	23.38±1.6	22.72±2.25	23.37±1.44

BG 2 W	4.72±0.47	23.23±1.74	23±0.76	22.87±2.07	22.77±1.91	23.5±1.97
BG 3 W	4.77±0.52	23.85±2.08	21.73±1	20.13±1.97	23.9±2.01	23.57±2.25
BG 4 W	4.88±0.29	24.8±1.56	22.03±1.58	21.13±0.94	25.17±1.19	24.15±1.5
BG 5 W	5.05±0.3	22.37±1.52	20.38±1.43	19.33±1.24	24.32±1.94	24.53±2.28
HR 3 d	18.33±5.97	9.03±3.61	10.76±3.89	8.64±1.64	12.34±2.59	14.1±3.16
HR 7 d	47.43±6.44	13.3±4.33	16.87±4.37	15.72±3.66	33.28±5.68	22.11±7.25
HR 10 d	68.06±7.57	24.01±3.89	25.92±2.43	24.54±7.66	47.49±4.47	49.41±3.78
HR 14 d	90.89±2.62	56.58±2.8	64.45±6.48	62.14±5.67	78.26±1.79	78.6±5

BG : blood glucose; HR: healing rates
